# Microcystins Induces Vascular Inflammation in Human Umbilical Vein Endothelial Cells via Activation of NF-κB

**DOI:** 10.1155/2015/942159

**Published:** 2015-05-06

**Authors:** Jun Shi, Jie Zhou, Min Zhang

**Affiliations:** ^1^Key Laboratory of Yangtze River Water Environment, College of Environmental Science and Engineering, Tongji University, Ministry of Education, Shanghai 200092, China; ^2^Department of Stomatology, Changzheng Hospital, Second Military Medical University, Shanghai 200003, China; ^3^Division of Cardiology, Tongren Hospital, School of Medicine, Shanghai Jiao Tong University, 1111 Xianxia Road, Shanghai 200336, China

## Abstract

Microcystins (MCs) produced by toxic cyanobacteria cause serious water pollution and public health hazard to humans and animals. However, direct molecular mechanisms of MC-LR in vascular endothelial cells (ECs) have not been understood yet. In this study, we investigated whether MC-LR induces vascular inflammatory process in cultured human umbilical vein endothelial cells (HUVECs). Our data demonstrated that MC-LR decreased HUVECs proliferation and tube formation and enhanced apoptosis. MC-LR also induced intracellular reactive oxygen species formation (ROS) in HUVECs. The MC-LR directly stimulated phosphorylation of NF-*κ*B. Furthermore, MC-LR also increased cell adhesion molecules (ICAM-1 and VCAM-1) expression in HUVECs. Taken together, the present data suggested that MC-LR induced vascular inflammatory process, which may be closely related to the oxidative stress, NF-*κ*B activation, and cell adhesion molecules expression in HUVECs. Our findings may highlight that MC-LR causes potential damage to blood vessels.

## 1. Introduction

Eutrophication of aquatic environments promoted to extreme cyanobacteria proliferation. The cyanobacteria metabolized much of toxin including microcystins (MCs), which were the most widely spread [1, 2]. In 2010, Wuxi (in Jiangsu, China) had an outbreak of a water crisis, because of a massive bloom of the cyanobacteria* Microcystis* spp. which occurred in Lake Taihu, which was the third largest freshwater lake in China. More than two million people lacked clean water for at least a week [3]. Microcystins were composed of seven amino acids and cyclic heptapeptides. More than 80 variants of microcystin have been found until now, and microcystin-LR (MC-LR) was the mostly toxic. Knowledge of MC-LR toxicity is necessary, because of both potencies of its acute cytotoxicity and tumor-inducing activity in animals and humans [4]. Natural exposure of animals and humans to MC-LR is mostly related to drinking contaminated water [2]. MC-LR has a direct effect on human cells, predominantly hepatocytes. Moreover, toxicity was also reported in kidney, lung, and other cells of the human tissues [5]. Simultaneously, endothelial cells (ECs) may be the most subjected to MC-LR during recreational activity and also be an important additional risk. The MC-LR strong effects on both whole organs and single cells have been reported in several researches. However, the effects of MC-LR on the EC have not been examined. When ECs have injury and dysfunction, many vascular diseases like metabolic syndrome happen. Vascular endothelial integrity was influenced by ECs proliferation and apoptosis, which maintained blood vessel function [6]. Therefore, whether MC-LR injured vessel functions via effecting ECs proliferation and apoptosis may be worth studying.

Nowadays, more and more evidences implied that MC-LR leads to damage in liver and kidney via oxidative stress [7]. The metabolism of MC-LR in animal model induced overexpression of reactive oxygen species (ROS), such as superoxide radical (O^2−^) and hydrogen peroxide (H_2_O_2_). In physiological conditions, ROS production in tissues could be efficiently scavenged by the antioxidant enzymes and glutathione- (GSH-) related enzymes, such as catalase (CAT) and superoxide dismutase (SOD). But overexpression of ROS could change endothelial function by many mechanisms, like peroxidation of membrane lipids and activation of NF-*κ*B [8]. Moreover, the vascular disorders induced more adhesion molecules, such as vascular cell adhesion molecule 1 (VCAM-1) and intracellular adhesion molecule 1 (ICAM-1), were thought to play an important role in controlling the pathological process of ECs inflammation [9]. However, the effect of MC-LR cytotoxic activity induced oxidative stress was not cleared in vascular ECs yet.

In this study, we treated human umbilical vein endothelial cells (HUVECs) with MC-LR to detect the precise effects on ECs proliferation, apoptosis, and tube formation. The possible signaling mechanisms by which MC-LR exerts its effects were also characterized. Our studies demonstrated for the first time that MC-LR induces vascular inflammation in HUVECs via activation of NF-*κ*B. MC-LR could also induce the expression of CAM-1 and VCAM-1, which were associated with the inflammatory process. All of these activated pathways may contribute to the vascular inflammatory action of MC-LR. Our findings reported that upregulation of ROS and activation of NF-*κ*B signaling is a critical mode of action of MC-LR for vascular inflammation.

## 2. Materials and Methods

### 2.1. Chemicals and Reagents

Microcystin-LR (purity 95%) was purchased from the Institute of Hydrobiology, Chinese Academy of Sciences, and protected from light until use. RPMI 1640 medium and fetal bovine serum (FBS) were obtained from Gibco (Grand Island, NY, USA). The cell lysis buffer (RIPA) and electrochemiluminescence (ECL) kit were purchased from Beyotime (Beijing, China). All antibodies, including primary and horseradish peroxidase-conjugated secondary antibodies, were purchased from Cell Signaling Technology (Beverly, MA, USA). FITC-conjugated phalloidin was purchased from Sigma (St. Louis, MO, USA). All other chemicals and reagents of the highest grade were purchased from commercial sources.

### 2.2. Human Umbilical Vein Endothelial Cells (HUVECs) Culture

Primary HUVECs were obtained from Allcells (Shanghai, China) and cells in passages 3–5 were used in this study. Cells were grown in an RPMI 1640 medium containing 20% fetal bovine serum (Gibco, Grand Island, NY, USA), 60 *μ*g/mL of endothelial cell growth supplement (BD, San Diego, CA), and 100 U/mL of penicillin with 100 *μ*g/mL of streptomycin (Gibco, Grand Island, NY, USA).

### 2.3. Cell Viability Assay

The viability of HUVECs upon treatment of MC-LR was measured by a colorimetric MTT assay. Briefly, HUVECs (2 × 10^5^ cells/well) were seeded on 24-well plates and cultured in RPMI 1640 medium containing 20% FBS for 24 h. After starvation in serum-free media for 12 h, HUVECs were treated with MC-LR (0–40 *μ*M) induced cells or an isovolumetric solvent control (0.1% DMSO) for 24 or 48 h. The cell number was measured using a colorimetric assay based on the ability of mitochondria in viable cells to reduce MTT. The cell number index was calculated as the absorbance of treated cells/control cells × 100%.

### 2.4. Apoptosis Analysis

HUVECs apoptosis was determined with the annexin V-FITC/propidium iodide assay. HUVECs (2 × 10^5^ cells/well) were seeded into 6-well plates. Then the cells were treated with MC-LR (0–40 *μ*M) for 24 h. They were then harvested, washed, and resuspended in PBS. Apoptotic cells were determined with an annexin V-FITC apoptosis detection kit (BD Biosciences, USA) according to the manufacturer's protocol. Apoptosis data were determined by using FlowJo (FlowJo, Ashland, OR, USA).

### 2.5. Endothelial Cell Transwell Migration Assay

The chemotactic motility of HUVECs was determined using a transwell migration assay (BD Biosciences) with 6.5 mm diameter polycarbonate filters (8 mm pore size). Briefly, the RPMI 1640 medium containing 10 ng/mL VEGF (500 *μ*L) was placed into the bottom chambers. HUVECs (5 × 10^4^) were incubated with MC-LR (0–40 *μ*M) in RPMI 1640 medium containing 0.5% FBS for 3 hours at 37°C before seeding into the upper chambers. After 8 h of incubation, nonmigrated cells in the upper chamber were removed. The migrated cells were fixed with 4% paraformaldehyde and stained with 1% calcein-AM. Images were taken using an Olympus microscope. Migrated cells in four random fields were quantified by manual counting. Three independent experiments were performed.

### 2.6. Endothelial Cell Capillary-Like Tube Formation Assay

The tube formation assay was conducted as follows. Briefly, each well of a 96-well plate was coated with 80 *μ*L BD Matrigel and incubated at 37°C for polymerization. HUVECs were trypsinized and suspended in RPMI 1640 medium containing 10 ng/mL VEGF (500 *μ*L) medium. Various concentrations of MC-LR (0–40 *μ*M) were added to the cells for 1 h at 37°C before seeding. Cells were then plated onto the Matrigel layer at a density of 2 × 10^4^ cells per well for 24 h. Cells with tube networks were visualized an Olympus microscope. Three independent experiments were performed.

### 2.7. Measurement of Intracellular ROS

Starved HUVECs (2 × 10^5^ cells/well) were loaded with DCF-DA (20 *μ*M) and MitoSOX dye, respectively. After treatment with MC-LR (0–40 *μ*M) or a solvent control for 24 h, cells were washed with PBS and then detached using trypsin. Levels of intracellular ROS were detected. All experiments were repeated at least four times to ensure reproducibility. For evaluating the contribution of NADH oxidase (NOX) and mitochondria on ROS production induced by MC-LR* in vitro*, cells were pretreated for 24 h with 40 *μ*M MC-LR and for 1 h with 500 nM of diphenyleneiodonium chloride (DPI; Sigma Aldrich, USA), a NOX inhibitor, or 500 nM of the mitochondria-targeting antioxidant Mito-Tempo (Sigma Aldrich, USA). Afterwards, total ROS produced by cells were measured as described above for the DCFDA probe.

### 2.8. SDS-Polyacrylamide Gel Electrophoresis (PAGE) and Western Blot Analysis

The HUVECs were treated with MC-LR (40 *μ*M) for 24 h. Western blot analyses were performed as lysates from each sample (40 *μ*g) were mixed with 6x sample buffer (0.35 M Tris, 10% w/v SDS, 30% v/v glycerol, 0.6 M DTT, and 0.012% w/v bromophenol blue, pH 6.8) and heated to 95°C for 5 min. Proteins were separated by electrophoresis and transferred onto polyvinylidene difluoride (PVDF) membranes for pNF-*κ*B, VCAM-1, ICAM-1, and TNF-*α*. The membranes were blocked with 5% nonfat milk in TBS-0.1% Tween 20 and sequentially incubated with primary antibodies and HRP-conjugated secondary antibodies, followed by enhanced chemiluminescence (ECL) detection. Data of specific protein levels are presented as relative multiples in relation to the control.

### 2.9. Statistical Analysis

Differences between treatments were assessed by one-way ANOVA followed by a Tukey test (Bartlett test *p* > 0.05) to compare mean of treatments with controls. Results are given as mean ± standard error of mean.

## 3. Results

### 3.1. MC-LR Decreased HUVECs Proliferation

**Figure 1:**
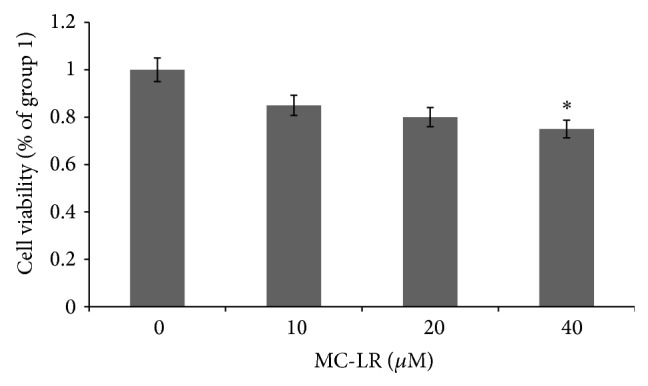
The MC-LR inhibited proliferation in HUVECs. Data are expressed as the mean values ± standard deviation of at least three independent experiments. Representative microphotographs are shown. ^∗^ *p* < 0.05 versus control (*n* = 3 independent experiments).

To investigate the role of MC-LR on HUVECs proliferation, it was observed that MC-LR at a concentration (40 *μ*M) can significantly decrease HUVECs proliferation for 24 h (Figure 1). Considering that the maximum effect appeared when the MC-LR concentration was 40 *μ*M; 40 *μ*M MC-LR was chosen for the following experiments. These data taken together suggested that MC-LR effectively reduced HUVECs proliferation.

### 3.2. MC-LR Induced HUVECs Apoptosis

**Figure 2:**
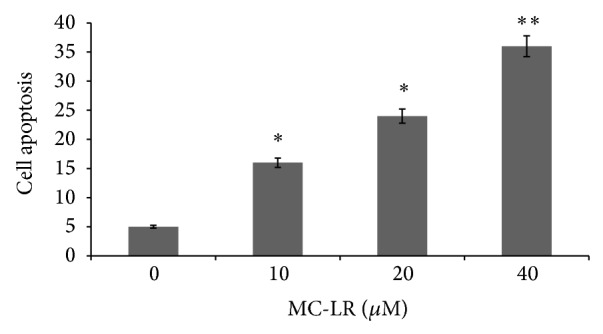
The MC-LR inhibited apoptosis in HUVECs. Data are expressed as the mean values ± standard deviation of at least three independent experiments. Representative microphotographs are shown. ^∗^ *p* < 0.05 and ^∗∗^ *p* < 0.01 versus control (*n* = 3 independent experiments).

To determine whether MC-LR has a direct effect on HUVECs apoptosis, the cells were incubated with MC-LR (0–40 *μ*M) for 24 h. As demonstrated in the flow cytometry results, MC-LR effectively induced apoptosis in HUVECs at a dose of 10 *μ*M (Figure 2). The percentage of cells apoptotic increased from 5.1 ± 0.6% to 35.8 ± 3.8% after being exposed to 40 *μ*M MC-LR for 24 h.

### 3.3. MC-LR Potently Inhibits Migration of HUVECs

**Figure 3:**
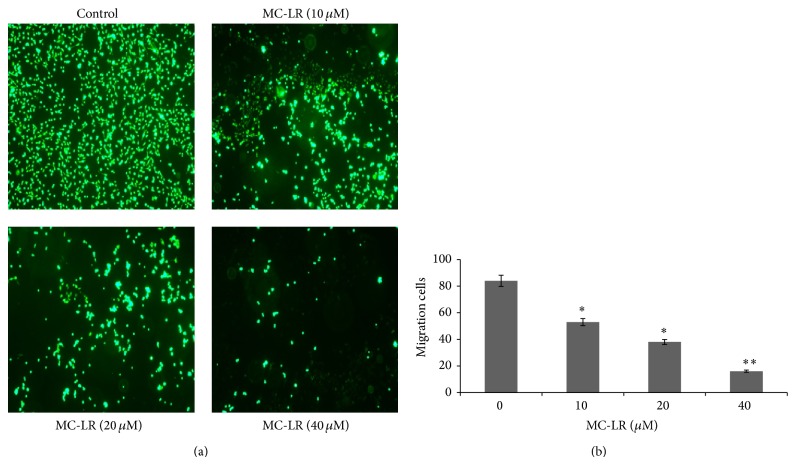
The MC-LR inhibited migration of HUVECs was detected by transwell assay. Representative microphotographs are shown. ^∗^ *p* < 0.05 and ^∗∗^ *p* < 0.01 versus control (*n* = 3 independent experiments).

The endothelial cell migration is an essential process in angiogenesis. The effects of MC-LR on the chemotactic motility of HUVECs were also detected with transwell migration assay (Figure 3(a)). Compared with basal medium, RPMI 1640 medium with VEGF triggered cell migration, but this effect was dose-dependently inhibited by MC-LR. The minimal effective action of MC-LR was shown at 10 *μ*M (Figure 3(b)). In the Boyden chamber assay, 40 *μ*M MC-LR indicated significant inhibition of endothelial cell motility, demonstrating that inhibition cell migration was an important aim of MC-LR effect (Figure 3(b)).

### 3.4. MC-LR Inhibits Capillary-Like Structure Formation of HUVECs

**Figure 4:**
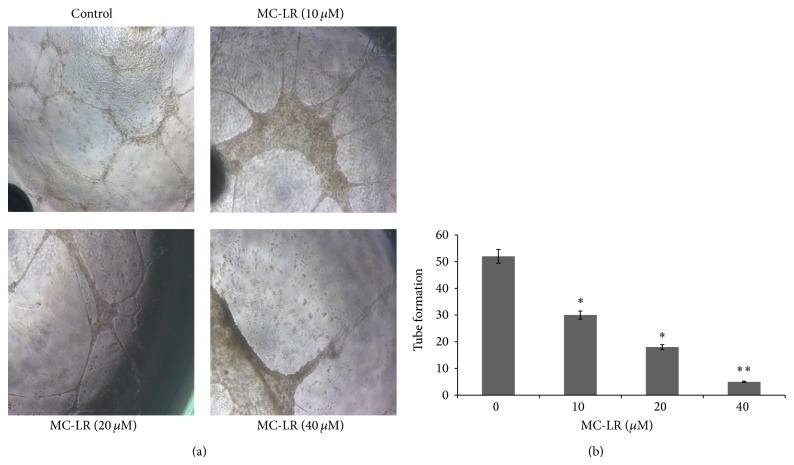
The MC-LR inhibited tube formation of HUVECs was detected by angiogenesis assay. ^∗^ *p* < 0.05 and ^∗∗^ *p* < 0.01 versus control (*n* = 3 independent experiments). The data were shown for three times. Scale bars, 200 *μ*m.

Although angiogenesis is a complex process, tube formation is one of the key steps. To investigate the potential effects of MC-LR (0–40 *μ*M) on HUVECs tube formation, we used a tube formation assay. The HUVECs were stimulated with VEGF, and then seeded on the matrigel, the tube formation was exhibited Figure 4(a). However, tube formation was inhibited by MC-LR in a dose-dependent way (Figure 4(b)). The MC-LR indicated a similar degree of potency for inhibition tube formation as shown in the HUVECs proliferation and migration assays.

### 3.5. MC-LR Induced ROS in HUVECs

**Figure 5:**
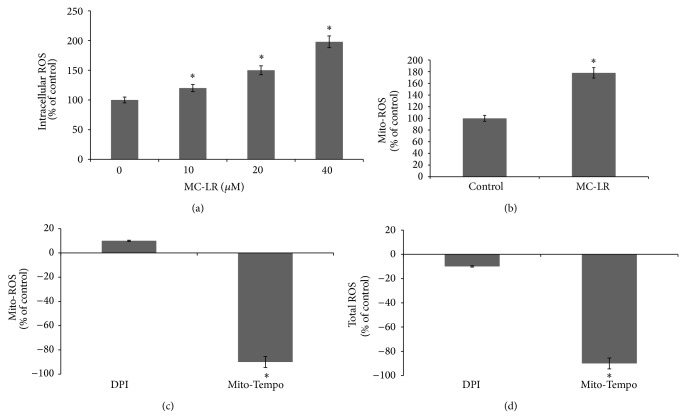
The MC-LR induced ROS expression on HUVECs was detected by DCFH-DA probe assay (a). The mitochondrial ROS in the control and MC-LR treated groups were measured by the DCFH-DA probe (b). The mean change of mitochondrial ROS (c) and total ROS (d) in MC-LR exposure HUVECs treated with DPI or Mito-Tempo. Representative microphotographs are shown. ^∗^ *p* < 0.05 versus control (*n* = 3 independent experiments).

The DCFDA (nonfluorescent in a resting state but fluorescent upon activation by ROS) was used to determine the efficiency of MC-LR (0–40 *μ*M) induced ROS formation in HUVECs. In our study, MC-LR induced ROS formation concentration-dependently as compared to resting (untreated) cells (Figure 5(a)). The overexpression ROS could influence cell-cycle progression, cell migration, and growth factor signaling in many of normal cell types. At first, we detected the ROS generation induced by MC-LR (Figure 5(a)). The intracellular ROS accumulated in spite of the catalytic action of NOX and/or the mitochondrial respiratory chain. By using MitoSOX probe, we found that mitochondrial ROS was notably increased in HUVECs treated with MC-LR (40 *μ*M) compared with the untreated cells (Figure 5(b)). As shown in Figure 5(b), MC-LR (40 *μ*M) induced twice total ROS formation compared to the control, whereas Mito-SOX exhibited 90% increase in total ROS compared to the control. To investigate the involvement of NOX in ROS formation induced by MC-LR (40 *μ*M), the cells was exposed to DPI. DPI at the dose used in our experiment (500 nM) was found to block NOX enzymes without affecting mitochondrial ROS production. As shown in Figures 5(c) and 5(d), the DPI did not reduce the mitochondrial ROS and total ROS production. These results indicated that mitochondria, not NOX, are responsible for the ROS formation induced by MC-LR.

### 3.6. Effect of MC-LR on NF-*κ*B Activation

**Figure 6:**
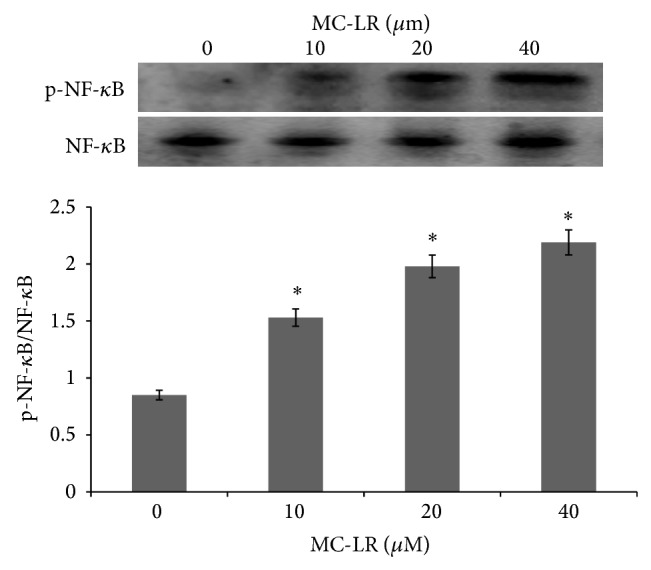
The MC-LR induced the expression of NF-*κ*B protein in HUVECs.

The ROS overexpression has been shown to activate various pathways including NF-*κ*B in cultured cells. In this study, it is proposed that the increased ROS production in MC-LR-induced HUVECs may partially cause the activation of NF-*κ*B. Therefore, we measured whether MC-LR (0–40 *μ*M) induced NF-*κ*B activation in HUVECs. Western blotting data showed that there was an increased expression of NF-*κ*B protein in HUVECs treated with MC-LR (Figure 6).

### 3.7. Effect of MC-LR on Endothelial Adhesion Molecules

**Figure 7:**
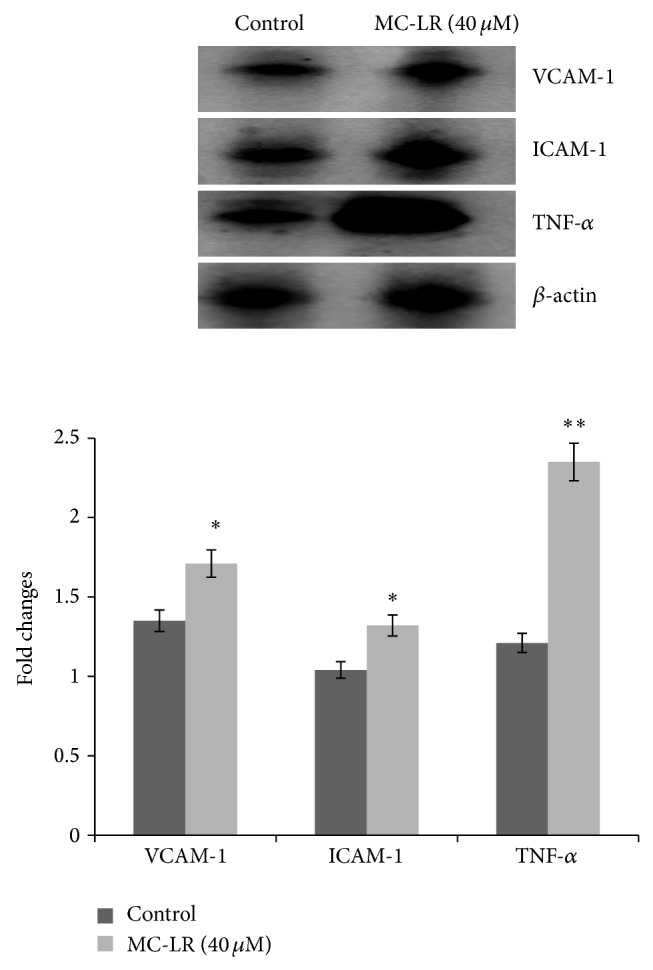
The MC-LR induced the expression of VCAM-1, ICAM-1, and TNF-*α* protein in HUVECs.

To investigate whether MC-LR induces expression of VCAM-1 and ICAM-1 in HUVECs, we cultured HUVECs at MC-LR (40 *μ*M) for 24 h. Immunoblot analysis showed that stimulation of HUVECs with MC-LR increased the production of VCAM-1 and ICAM-1 (Figure 7).

### 3.8. MC-LR Inducted TNF-*α* Expression

The TNF-*α* is involved in the inflammatory response, apoptosis, and cell proliferation. Additionally, TNF-*α* could regulate the expression of various other cytokines. So we analyzed whether the MC-LR (40 *μ*M) inducted the TNF-*α* expression. The results showed a significant induction of TNF-*α* expression which occurred after 24 h exposure to 40 *μ*M MC-LR (Figure 7).

## 4. Discussion

Earlier studies have confirmed the nephrotoxicity and hepatotoxicity of MC-LR [10]. In this study, we evaluated the effect of MC-LR on multiple steps of endothelial cell function. In HUVECs viability assay, the higher concentration of MC-LR (40 *μ*M) consistently exhibited potent antiproliferative activities. The HUVECs apoptosis enhanced from 10 *μ*M MC-LR because of cytotoxicity, but the proliferation of HUVECs did not change at the same concentration. It was important in the case of short-term exposure to the MC-LR. In the past researches, the cell proliferation inhibition led to impairing wound healing and angiogenesis; the MC-LR maybe delayed the healing process but did not complete destruction. However, pretreated HUVECs in the presence of 10 *μ*M MC-LR for 48 hours decreased cells proliferation (data not shown). The reason maybe was the more uptake of MC-LR that occurred. However, more researches should be needed to confirm the effect of MC-LR inside the cells. The motile activity of HUVECs is an important feature of angiogenesis. We observed that MC-LR significantly influenced the migration of HUVECs at a range of concentrations (10–40 *μ*M). The lower concentrations MC-LR was observed drastically regulated the HUVECs migration activity. The MC-LR inhibited endothelial cell migration and tube formation at concentrations of 10–40 *μ*M. However, the endothelial cell proliferation was not changed at the lower concentration (10 *μ*M). Meanwhile, The percentage of apoptotic cells increased about 10% after MC-LR treated (10 *μ*M). In addition, the percentage of migration cell decreased about 30% after MC-LR treated (10 *μ*M). To some degree, the results suggested an important target of MC-LR was the molecules that participated in cells motility. This suggested that MC-LR toxicity might account at least in part for decreasing motile activity of HUVECs. Our study was undertaken to know the mechanisms by which MC-LR impairs effects on human vascular. For the first time, we have shown that MC-LR induced vascular inflammation via increasing of ROS, CAMs, and NF-*κ*B in primary cultured HUVECs.

The endothelial dysfunction could obviously enhance the risk of all forms of cardiovascular complications including critical limb ischemia and foot ulcers. However, no previous studies have evaluated how MC-LR directly changes endothelial cells function. In our study, it was demonstrated that MC-LR can directly decrease HUVECs functions. Moreover, regulating endothelial cells proliferation and apoptosis could significantly affect vascular endothelial integrity and sustain endothelium homeostasis. So the MC-LR decreased HUVECs proliferation and induced apoptosis, which may suggest blood vessel function damage.

This study also examined whether MC-LR affects oxidative stress of HUVECs, as several lines of evidence showed that MC-LR induced ROS production. All results revealed that MC-LR significantly increased the ROS production (Figure 5). The ROS production has been linked closely with inflammatory responses [11, 12]. Both of the ROS production and inflammation were potential mediators of vascular diseases. In our results, MC-LR induced increment of the cellular ROS production may indicate that the MC-LR potential induces vascular inflammation.

Another interesting finding of our study was the case that NF-*κ*B might be an aim of MC-LR induced vascular inflammation. The NF-*κ*B pathway could regulate the expression of genes encoding cell adhesion molecules [13]. Thus, improvement of approaches targeting this pathway could present a novel therapeutic tool for the treatment of many diseases [14]. NF-*κ*B bounded to the inhibitor protein I-*κ*B in normal condition. But when the cell was stimulated by cytokines or endotoxin, the phosphorylation of inhibitor *κ*B and the activation of NF-*κ*B happened [15]. This in turn induced the overexpression of cell adhesion molecules, chemokines and macrophage migration inhibitory factor, and matrix metalloproteinases-1 and -9, which could regulate cell apoptosis and proliferation [16]. The results of this study demonstrated that MC-LR induced NF-*κ*B activation in HUVECs.

In our study, we also detected the expression of ICAM-1, VCAM-1, and TNF-*α*, which implied a signal for the HUVECs activation. These cell adhesion molecules participated in cell adherence and inflammatory responses. Increased expression of ICAM-1, VCAM-1, and TNF-*α* was observed in MC-LR treated HUVECs, and further research indicated that the level of TNF-*α* was more strongly regulated by high content MC-LR treated. These data showed a possible depiction of increased vascular permeability in MC-LR treated HUVECs. Thus, the ICAM-1 and VCAM-1 expression proved useful evidences that the MC-LR induced vascular inflammation.

Accumulating evidences indicated that the inflammatory cytokine TNF-*α* plays a key role in the vascular function lesions and the following development of vascular disease. Epidemiological studies have shown that TNF-*α* expression was extremely increased in the plasma and arteries of humans with vascular complications. Moreover, TNF-*α* overexpression has been estimated as an injury-related signal in many researches involving inflammation. Furthermore, TNF-*α* has been shown to induce the expression and release of many adhesion molecules and chemokines involved in the inflammatory response. Thus, all the data including TNF-*α* overexpression showed more evidences that the MC-LR induces vascular inflammation.

## 5. Conclusion

In conclusion, the most major findings of our study proved that MC-LR enhances inflammatory response in HUVECs for the first time. In this study, we observed that MC-LR induced VCAM-1 and ICAM-1 expression in HUVECs through NF-*κ*B pathway. Therefore, this study suggested that MC-LR could be very harmful in the vascular inflammatory process and its precise mechanism needs to be further studied.
